# Does the “delayed start” protocol with gonadotropin-releasing hormone antagonist improve the pregnancy outcome in Bologna poor responders? a randomized clinical trial

**DOI:** 10.1186/s12958-018-0442-y

**Published:** 2018-12-28

**Authors:** Mahnaz Ashrafi, Arezoo Arabipoor, Azar Yahyaei, Zahra Zolfaghari, Firouzah Ghaffari

**Affiliations:** 1 Department of Endocrinology and Female Infertility, Reproductive Biomedicine Research Center, Royan Institute for reproductive biomedicine, ACECR, P.O Box: 19395-4644, No 12, East Hafez Avenue, Banihashem Street, Resalat highway, Tehran, Iran; 2 Obstetrics and Gynecology Department, Faculty of Medicine, Iran University of Medical Science, Tehran, Iran; 3 Department of Epidemiology and Reproductive Health, Reproductive Epidemiology Research Center, Royan Institute for Reproductive Biomedicine, ACECR, Tehran, Iran

**Keywords:** Poor responders, GnRH antagonist, Conventional protocol, Delayed start protocol, Bologna criteria

## Abstract

**Background:**

Recently, a novel approach with delaying the start of controlled ovarian stimulation along with gonadotropin-releasing hormone (GnRH) antagonist pretreatment for 7 days after estrogen priming for further suppression of endogenous follicle stimulating hormone (FSH) during the early follicular phase, resulting in more FSH-responsive follicles and thus improving synchronous follicular development was introduced. Two clinical trials have examined this strategy and reported controversial results. This study aimed to compare the effect of delayed-start GnRH antagonist protocol and standard GnRH antagonist in patients with poor ovarian response (POR) undergoing in vitro fertilization (IVF)/ *intracytoplasmic sperm injection* (ICSI).

**Methods:**

This randomized clinical trial was conducted at infertility department of Royan Institute from January 2017 to June 2018. Poor ovarian response was defined according to the Bologna criteria. The eligible women were randomly allocated into an experimental and control groups. In experimental group, patients received delayed-start GnRH antagonist protocol with estrogen priming followed by early follicular-phase GnRH antagonist treatment for 7 days before ovarian stimulation with gonadotropin and in control group, patients treated with estrogen priming antagonist protocol. IVF/ICSI outcomes were compared between groups.

**Results:**

*Among* all the 250 patients examined 156 women were eligible for study and finally 120 patients were allocated to intervention (*n* = 60) and control (*n* = 60) groups. Demographic characteristics and hormonal profiles of the patients did not differ between groups. The statistical analysis showed that there were significant differences between groups regarding the total dose of used gonadotropins (*P* < 0.001), stimulation duration (*P* < 0.001), number of retrieved oocytes (*P* = 0.01) and top quality embryo (P < 0.001) and also cancellation (*P* = 0.002) and fertilization rates (*P* = 0.002).

**Conclusion:**

On the basis of present results the delayed-start protocol in poor responders can improve the fertilization rate and quality of embryos and reduce the cycle cancellation but have no significant effect on clinical pregnancy rate; however, larger randomized clinical trials are required to compare it with other protocols.

**Trial registration:**

NCT, NCT03134690. Registered 1 May 2017 - Retrospectively registered, http://www.clinicaltrial.gov/ NCT03134690.

## Background

One of the principal steps to obtain the favorable success is still related to the number of retrieved oocytes after hormonal stimulation by gonadotropins in combination with gonadotropin releasing hormone (GnRH) analogues [1]. In the patients with “poor ovarian response” (POR) diagnosis, the limited number of obtained oocytes remains the main obstacle in optimizing the pregnancy rates, so it is a frustrating event for both patients and clinicians which is associated with high cycle cancellation rate and poor pregnancy outcomes [1, 2]. Different types of controlled ovarian stimulation (COS) regimens have been reported to improve the cycle outcomes in POR patients, but still there is no consensus on the ideal COS protocol in such patients [1–4].

Alterations in intra ovarian factors or gonadotropin receptor regulation [5], a shortened follicular phase with limited ability to recruit a cohort of follicles and different sensitivity of early antral follicles to follicle stimulating hormone (FSH) [6, 7] are presented as possible etiologies for a poor response [8]. It is possible that some antral follicles are able to respond to the lower amounts of FSH better than others depending on their inherent sensitivity to FSH. Therefore start to develop during the late luteal phase, accentuating size discrepancies observed during the first days of the subsequent cycle and leading to asynchronous growth with COS (unclear) [8]. Recently, Cakmak et al., in a retrospective study introduced a novel approach with delaying the start of COS along with GnRH antagonist pretreatment for 7 days after estrogen priming for further suppression of endogenous FSH during the early follicular phase, resulting in more FSH-responsive follicles and thus improving synchronous follicular development [8]. Also, two clinical trials have examined this strategy and reported controversial results [9, 10]. The present randomized clinical trial was designed to compare the efficacy of delayed-start GnRH antagonist protocol versus GnRH antagonist protocol in patients with poor ovarian response diagnosis on the basis of Bologna criteria.

## Methods

This randomized clinical trial was conducted at infertility department of Royan Institute from January 2017 to June 2018. The trial protocol was approved by the Institutional Review Board and Ethics Committee of Royan Institute (Ethics code: IR.ACECR.ROYAN.REC.1394.121). The eligible patients signed the informed consent. Patients with poor ovarian response undergoing IVF/ICSI and fresh embryo transfer (ET) cycles were evaluated. Poor ovarian response was defined according to the Bologna criteria and existence of at least two of the following criteria: 1) a previous history of POR (retrieved oocytes ≤3) in a conventional stimulation protocol, 2) advanced maternal age (≥40 years) or any other risk factors for POR (e.g. a history of ovarian surgery) and 3) abnormal ovarian reserve test (i.e. antral follicle count (AFC) < 5 follicles or anti-Mȕllerian hormone (AMH) < 1.1 ng/ml). The exclusion criteria were premature ovarian failure (basal follicle stimulating hormone (FSH) above 20 IU/l or no antral follicle in ultrasound examination), donor/recipient treatments, metabolic or endocrine disorders including hyperprolactinoma and hypo/hyperthyroidism, endometriosis, body mass index > 30 kg/m^2^, and azoospermic male partner. A minimum of 2 or more month’s interval from the previous ovarian stimulation was considered to prevent any potential source of error.

Block randomization method was designed by a fellow epidemiologist using Stata software version 13 and the number of blocks were 4. The random allocation list for patients was only accessible to the epidemiologist. In order to random allocation concealment, only the methodologist was aware of the design of the code. When the doctor confirmed patient’s eligibility, the methodologist provided the doctor with the envelope. The group was selected based on the type of group mentioned in the envelope. The outcome evaluators were also blinded to the random allocation process and type of treatment. Data analysis was performed by a statistician who was also unaware of all processes performed.

On second day of menstrual cycle, the eligible patients were randomly allocated into either delayed start or routine GnRH-antagonist stimulation protocol in a 1:1 ratio. A flexible regimen of GnRH-antagonist was used for all study participants. The serum estradiol (E_2_) concentrations < 60 pg/mL and absence of ovarian cysts < 10 mm diameter on vaginal ultrasound scans on cycle day 2 were used to define ovarian quiescence. The baseline serum FSH and luteinizing hormone (LH) levels were also measured at initial assessment before gonadotropin stimulation. All patients received estrogen priming (Estraval®, 2 mg*,* Aburaihan Co., Tehran*,* Iran) starting one week after LH surge and continued until mensturation and prior to ovarian stimulation. In the delayed-start protocol, baseline ultrasounds were performed on cycle day 2 and after the completion of GnRH antagonist pretreatment to identify the absence of ovarian cyst or lead follicle > 10 mm. In conventional antagonist protocol (control group), ovarian stimulation with gonadotropins was started on day 2 of menstrual cycle. In the delayed-start protocol (experimental group), ovarian stimulation was started after 7 days of GnRH antagonist pretreatment (Cetrotide®, 0.25 mg cetrorelix acetate*,* Serono, *Inc*). In both protocols, 300 IU FSH (Gonal - F®, Serono Laboratories Ltd., Geneva, Switzerland) and 150 IU human menopausal gonadotrophins (HMG) (Menopur; Ferring) were used for ovarian stimulation. The serial vaginal ultrasound (sonographic device: Phillips, affinity 70) and measurements of serum estradiol (E_2)_ level were used to assess follicular maturation. The dosage of gonadotropins was adjusted according to the ovarian response. In both groups, when follicle(s) ≥13 mm were observed, the GnRH antagonist, cetrorelix (Cetrotide ®, Serono International, Geneva, Switzerland), 0.25 mg/day was started subcutaneously and continued until the day of triggering of ovulation. Progesterone and E_2_ levels were evaluated in serum on day of human chorionic gonadotropin (hCG) administration. When at least one follicles measuring ≥18 mm in diameter and serum E_2_ concentration ≥ 500 pg/mL were observed, the final stage of oocyte maturation was induced by two pre-filled syringes of *recombinant human chorionic gonadotropin* (rhCG) (Ovitrelle®, 250 μg/0.5 ml, Merck, Serono, Inc). If these criteria have not been achieved after 10–12 days stimulation, the cycle has been cancelled for inadequate response. Transvaginal ultrasound-guided oocyte retrieval was performed 34–36 h after oocyte triggering. After stripping the cumulus cells, intracytoplasmic sperm injection (ICSI) was done with ejaculated sperm to metaphase II (MII) oocytes in all cycles. ICSI was performed in all cases to prevent infrequent cases of fertilization failures with conventional IVF. Embryos were cultured in a commercially available culture medium until the day of transfer. The obtained embryos at cleavage stage were replaced by an embryo transfer catheter (Guardia™, Access ET Catheter, Cook Medical), 2 or 3 days after oocytes retrieval. Embryo quality was determined according to the number and regularity of blastomeres and the degree of embryonic fragmentation that has been explained previously [11]. All patients received luteal phase support in the form of 400 mg vaginal progesterone suppository twice daily (Cyclogest® (400 mg), Actavis, Barnstaple, UK) starting on the evening of the oocyte retrieval and it was continued for 10 weeks in cases with positive pregnancy test. A serum ß-hCG analysis was done 14 days after ET, and the clinical pregnancy (presence of gestational sac with heartbeat) was determined by ultrasound scan 14 days later.

The main outcomes were the fertilization and cycle cancellation rates, the numbers of retrieved and MII oocytes, obtained and top quality embryos respectively. The secondary outcomes were the total gonadotropins dose, duration of ovarian stimulation, endometrial thickness, implantation and clinical pregnancy rates.

### Statistical analysis

According to pilot study and by using G*Power software (version 3.1.9.2) with considering the effect size of 0.10, α = 0.05, and 80% power for fertilization rate as primary outcome; 60 subjects were needed in each study group. The statistical analysis was carried out by using Statistical Package for the Social Sciences, version 20, SPSS Inc., Chicago, Illinois, USA (SPSS). The differences between two groups were analyzed using the independent t-test and Mann-Whitney U test for the normal and non-normal continuous variables respectively. The Chi- square test was applied for comparison of the categorical variables between groups. Descriptive data are presented as mean ± standard deviation (SD) or median (interquartile range) as indicated. The Statistical significance level was set at *p*-value < 0.05.

## Results

**Fig. 1:**
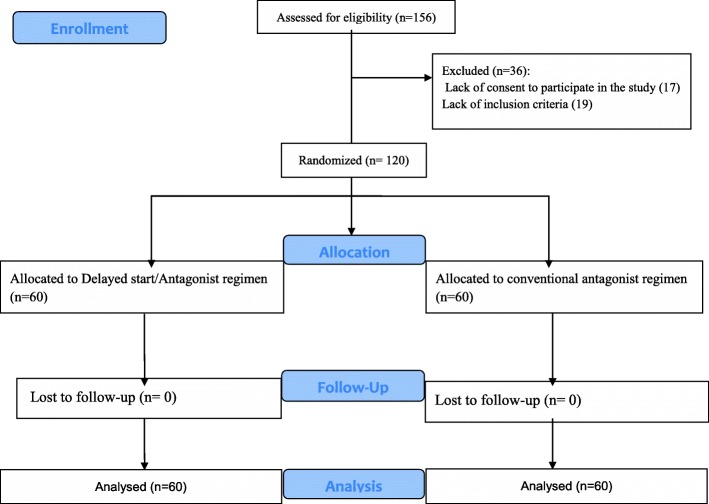
Recruitment follow-up and dropouts during study

According to Fig. 1, among 250 patients examined, 156 women were eligible for study and a total of 120 patients were allocated to intervention (*n* = 60) and control (n = 60) groups. Demographic information and hormonal profiles of the patients are presented in Table 1. According to this table, there were no significant differences between groups.

**Table 1:** Demographic and clinical characteristics of study participants in two groups

Variables	Control group (*N* = 60)	Delayed start group (*N* = 60)	*P* value
Female age (yr.)	39.5 ± 4.8	40.5 ± 4.48	0.2
Body mass index (kg/m^2^)	26.1 ± 3.2	26.8 ± 3.0	0.2
Duration of Infertility (yr.)	9.2 ± 6.7	7.9 ± 5.2	0.2
No. of couple with primary infertility n (%)	44 (73.3)	42 (70.0)	0.8
Early follicular phase FSH (IU/L)	8.7 ± 3.3	8.0 ± 2.8	0.3
Early follicular phase LH (IU/L)	5.5 ± 4.2	6.5 ± 4.5	0.1
Early follicular phase AMH (ng/mL)	0.53 ± 0.31	0.55 ± 0.30	0.8
Serum level of TSH (IU/mL)	1.7 ± 0.8	1.9 ± 1.1	0.2
Serum level of Prolactin (ng/mL)	17.8 ± 10.0	17.8 ± 8.7	0.6
Antral follicle count	5.0 ± 1.8	4.7 ± 1.2	0.3
No. of previous failed cycles	1.5 ± 1.2	1.3 ± 1.0	0.1
Subgroups of POR according to Bologna criteria n, (%)			0.2
Subgroup A	0	0	
Subgroup B	18 (30)	19 (31.7)	
Subgroup C	18 (30)	10 (16.6)	
Subgroup D	24 (40)	31 (51.7)	

Descriptive data were presented as Mean ± SD. *P*-value≤0.05 was considered statistically significant

*No* number, *FSH* Follicle stimulating hormone, *LH* = Luteinizing hormone, *TSH* = Thyroid stimulating hormone, *AMH* Anti-müllerian hormone, *E*_*2*_ = estradiol, *P* = Progesterone

Subgroup A: (female age ≥ 40, and a previous poor response (cycle cancelled or ≤ 3 oocytes)

Subgroup B: female age ≥ 40 with an abnormal antral follicle count (AFC) < 7 and AMH level

Subgroup C: abnormal AFC < 7 and AMH < 1.1 ng/ ml with a previous poor response

Subgroup D: female age ≥ 40 with an AFC < 7 and AMH < 1.1 ng/ ml and previous poor response

Cycle outcomes are presented in Table 2. The dose of gonadotropins and the duration of the ovarian stimulation cycle in the delayed start group were significantly lower than the control group (*P* < 0.001). The number of retrieved oocytes, fertilization rate, number of good quality embryos, number of embryos transferred, and endometrial thickness on the day of HCG administration were significantly higher in the delayed start than the control group. The number of obtained embryos was also significantly higher in the delayed start than the control group (*P* = 0.02). The rate of cycle cancellation in the control group was 30% while no cycle cancellation was observed in the conventional group. However, there were not any statistically significant differences between groups in terms of embryo implantation, clinical pregnancy, and abortion rates.

**Table 2:** Comparison of stimulation and cycle outcomes in the two study groups

Variables^a^	Delayed start group (N = 60)	Control group (*N* = 60)	95% CI	*P* value
Total rFSH dose (IU)	2210.0 ± 559.4	2897.0 ± 952.0	(− 969.8–405.1)	< 0.001
Total hMG dose (IU)	1122.5 ± 272	1487 ± 486	(− 507.4–222.5)	< 0.001
Duration of stimulation (day)	9.3 ± 1.7	12.3 ± 3.6	(− 3.9–1.8)	< 0.001
No. of retrieved oocytes	3.2 ± 2.7	2.0 ± 2.1	(0.22–2.0)	0.01*
No. of metaphase II oocytes	2.5 ± 2.2	1.8 ± 1.9	(−0.06–1.4)	0.07
The cases with no oocyte result, *n* (%)	6 (10)	2 (3.4)	–	0.1
Cancellation rate (no response), *n* (%)	0 (0)	18 (30.0)	–	< 0.001
Fertilization rate	73.4 ± 36.3	42.6 ± 36.7	(15.4–46.0)	< 0.001*
No. of obtained embryos	2.3 ± 1.6	1.7 ± 0.8	(−0.07–1.2)	0.02*
No. of top quality embryo	1.5 ± 1.3	1.0 ± 0.7	(−0.06–1.0)	0.02*
The cases with no embryo result, *n* (%)	8 (13.3)	12 (20.0)	–	0.3
No. of embryos transferred	1.8 ± 0.9	1.7 ± 0.8	(−0.04–0.5)	0.7
Endometrial thickness at trigger (mm)	9.3 ± 1.2	8.7 ± 0.9	(0.12–0.96)	0.01*
Implantation rate	41.6 (33.3–50)	50 (50–50)	–	0.5
Clinical pregnancy/ ET (%)	4/46 (8.7)	2/28 (7.1)	–	0.8
Miscarriage rate/ET (%)	0 (0)	1 (3.5)	–	0.1

^a^Descriptive data were presented by mean ± standard deviation and median (interquartile range) as indicated. The binary variables were presented as number (percent). CI: confidence interval of the difference, *P-value ≤0.05 was considered statistically significant. *hMG* human menopause gonadotropin, *rFSH* recombinant follicle stimulating hormone, *No* number. *ET* embryo transfer

## Discussion

In the present study, we compared cycle and pregnancy outcomes in poor responders with early follicular GnRH antagonists pretreatment for 7 days after preceding late luteal estrogen priming and before the beginning of ovarian stimulation (delayed start protocol) with GnRH antagonists with estrogen priming without GnRH antagonists pretreatment. Our results showed higher number of retrieved oocytes and fertilization rate and also higher number of top quality embryos in delayed GnRH-antagonist protocol than the conventional group, although the rates of pregnancy and implantation were not significantly different between groups. It is of crucial importance that this protocol can reduce the rate of cycle cancellation.

A retrospective study demonstrated the positive effect of this strategy in antagonist protocol [8]. Similarly, Maged et al.; in a first randomized clinical trial on 160 patients with POR diagnosis showed that the delayed start protocol improved cycle outcome by reducing the total dose of used gonadotropin, improving estradiol levels (E_2_) and endometrial thickness on the day of hCG administration and increasing the total number of retrieved and mature oocytes [9]. However, Aflatoonian et al. in a randomized pilot study on 60 POR failed to show any significant difference between this new strategy and routine antagonist protocol [10]; although the non-significant results may relate to small sample size. In our study, the clinical pregnancy rate in both study and control groups were low (8.7% vs. 7.1%, respectively). In contrast, Maged et al. presented significant high pregnancy rate with delayed start protocol compared to conventional antagonist one (30% vs. 10%); meanwhile, it was 13.3% vs. 3.3% in Aflatoonian et al. study. This contradiction in pregnancy outcomes could be due to the differences in the sample size and the subgroups or phenotypes of POR patients among various studies. In both previous studies, the Bologna criteria were applied to include POR patients, but it was not clear which phenotypes or subgroups of the Bologna criteria. In present study, the majority of the patients were determined as subgroup C and D who had all three points of the Bologna criteria and so called as “expected poor responders”. Also, there is evidence that these subgroups (C and D) have poorer outcomes than other subtypes (A and B) [12].

In a recent clinical trial, Davar et al. evaluated 100 women with POR and compared delayed start protocol with GnRH antagonist with micro micro-dose flare-up GnRH agonist protocol. They found significant improvement in the number of retrieved and mature oocytes and implantation rate with delayed antagonist group; however, the fertilization, clinical pregnancy, and ongoing pregnancy rates were not significantly different between groups [13]. Considering that the studies comparing this new strategy in antagonist protocol with other standard protocols in patients with POR are very limited, it is interesting to suggest a clinical trial with large sample size to compare its cost-effectiveness with other protocols.

The beneficial effect of early follicular phase GnRH antagonist on increasing the number of retrieved oocytes and the size of antral follicles on day 8 of gonadotropins treatment and competence of retrieved oocytes in normal responder patients were reported in previous studies [14–16]; similarly, they found no significant positive effect on pregnancy rate in these patients. Also, a retrospective study on 65 poor responders who underwent E_2_/GnRH antagonist priming protocol found significantly better stimulation and pregnancy outcome in comparison with control group. It was concluded that E_2_/GnRH antagonist priming protocol could improve IVF outcomes in poor responders by suppressing endogenous FSH and preventing premature luteinization [17]. In POR patients, the high doses of used gonadotropins could had negative impact on endometrial receptivity; on the basis of the present findings and previous evidence [18, 19], we suggest freeze-all embryos strategy for overcoming the endometrium damaged induced by high dose of hormonal drugs used through this COS protocol in POR patients. In addition, regarding to significant reduction in the cycle cancellation rate in the recent COS protocol, it is possible to reduce the risk of an aneuploidy in these patients by proposing the accumulation of embryos and performing pre-gestational diagnosis and increased the probability of clinical pregnancy per embryo transfer.

In the present study, the ovarian stimulation with delayed start is associated with higher endometrial thickness on hCG day compared to routine protocol which may due to different E_2_ levels. However, there were no significant differences in implantation and clinical pregnancy rates between groups. Whereas, Maged et al. found increased clinical pregnancy rate along with increased endometrial thickness on hCG day in delayed start group when compared with control group [9]. It seems that endometrial thickness on hCG day could be effective on endometrial receptivity and pregnancy rate [20]; although the present study failed to find this relationship. The power of present study for comparing the implantation and clinical pregnancy rates between groups is limited, because these issues were our secondary outcomes; therefore we suggested further studies with larger sample size to evaluate pregnancy outcome as main objective.

Nevertheless, the present study has some limitations and some strength points that should be mentioned. The strength of present study was the randomized clinical trial methodology and selection of homogenous population on POR patients. Considering the low prevalence of POR, we achieved an appropriate sample size; however, the sample size of present study was lower than Maged et al. study and it could be a potential weakness.

## Conclusion

Delayed-start protocol can improve the fertilization rate and quality of embryos and prevent cycle cancellation but have no significant effect on clinical pregnancy rate. Larger prospective randomized studies are required to compare delayed antagonist with the conventional protocol and also other protocols such as stop GnRH agonist, mini-flare up and Shanghai (double mild stimulation in the same cycle).

## Abbreviations

AFC: Antral follicle count
COS: Controlled ovarian stimulation
E_2_: Estradiol
ET: Embryo transfer
FSH: Follicle stimulating hormone
GnRH: Gonadotropin-releasing hormone
hCG: Human chorionic gonadotropin
*HMG*: Human menopausal gonadotrophins
IVF/ICSI: In vitro fertilization/ Intra-cytoplasmic sperm injection
POR: Poor ovarian response
SD: Standard deviation
